# Digital Spatial Profiling Identifies the Tumor Periphery as a Highly Active Biological Niche in Clear Cell Renal Cell Carcinoma

**DOI:** 10.3390/cancers15205050

**Published:** 2023-10-19

**Authors:** Felix Schneider, Adam Kaczorowski, Christina Jurcic, Martina Kirchner, Constantin Schwab, Viktoria Schütz, Magdalena Görtz, Stefanie Zschäbitz, Dirk Jäger, Albrecht Stenzinger, Markus Hohenfellner, Stefan Duensing, Anette Duensing

**Affiliations:** 1 Molecular Urooncology, Department of Urology, University Hospital Heidelberg, Im Neuenheimer Feld 517, D-69120 Heidelberg, Germany; 2 Institute of Pathology, University Hospital Heidelberg, Im Neuenheimer Feld 224, D-69120 Heidelberg, Germany; 3 Department of Urology, University Hospital Heidelberg, and National Center for Tumor Diseases (NCT), Im Neuenheimer Feld 420, D-69120 Heidelberg, Germany; 4 Department of Medical Oncology, University Hospital Heidelberg, and National Center for Tumor Diseases (NCT), Im Neuenheimer Feld 460, D-69120 Heidelberg, Germany; 5 Cancer Therapeutics Program, UPMC Hillman Cancer Center, 5117 Centre Avenue, Pittsburgh, PA 15213, USA; 6 Department of Pathology, University of Pittsburgh School of Medicine, 200 Lothrop Street, Pittsburgh, PA 15213, USA; 7 Precision Oncology of Urological Malignancies, Department of Urology, University Hospital Heidelberg, Im Neuenheimer Feld 517, D-69120 Heidelberg, Germany

**Keywords:** renal cell carcinoma, intratumoral heterogeneity, spatial biology, tumor periphery, granzyme B

## Abstract

**Simple Summary:**

Clear cell renal cell carcinoma is characterized by a high degree of genomic intratumoral heterogeneity. The extent of spatial and functional heterogeneity, however, is much less understood. In the present study, digital spatial profiling identified the tumor periphery as a highly active biological niche with the upregulation of a number of proteins involved in tumorigenic signaling nodes. One of the most significantly upregulated proteins was granzyme B, which plays an important role in anti-cancer immune responses. Patients exhibiting such an overexpression in the tumor periphery had a significantly worse cancer-specific survival. It is possible that high granzyme B expression creates selection pressure for tumor cells to escape antitumoral host responses. Results from this study highlight the role of the tumor periphery as a hotspot for various signaling activities and tumor evolution. Our findings have important implications for the future development of predictive and prognostic biomarkers to improve patient stratification.

**Abstract:**

Clear cell renal cell carcinoma (ccRCC) is characterized by a high degree of intratumoral heterogeneity (ITH). Besides genomic ITH, there is considerable functional ITH, which encompasses spatial niches with distinct proliferative and signaling activities. The full extent of functional spatial heterogeneity in ccRCC is incompletely understood. In the present study, a total of 17 ccRCC tissue specimens from different sites (primary tumor, *n* = 11; local recurrence, *n* = 1; distant metastasis, *n* = 5) were analyzed using digital spatial profiling (DSP) of protein expression. A total of 128 regions of interest from the tumor periphery and tumor center were analyzed for the expression of 46 proteins, comprising three major signaling pathways as well as immune cell markers. Results were correlated to clinico-pathological variables. The differential expression of granzyme B was validated using conventional immunohistochemistry and was correlated to the cancer-specific patient survival. We found that a total of 37 proteins were differentially expressed between the tumor periphery and tumor center. Thirty-five of the proteins were upregulated in the tumor periphery compared to the center. These included proteins involved in cell proliferation, MAPK and PI3K/AKT signaling, apoptosis regulation, epithelial-to-mesenchymal transition, as well as immune cell markers. Among the most significantly upregulated proteins in the tumor periphery was granzyme B. Granzyme B upregulation in the tumor periphery correlated with a significantly reduced cancer-specific patient survival. In conclusion, this study highlights the unique cellular contexture of the tumor periphery in ccRCC. The correlation between granzyme B upregulation in the tumor periphery and patient survival suggests local selection pressure for aggressive tumor growth and disease progression. Our results underscore the potential of spatial biology for biomarker discovery in ccRCC and cancer in general.

## 1. Introduction

Clear cell renal cell carcinoma (ccRCC) is among the most lethal urological malignancies once metastatic. This tumor entity is characterized by a high level of intratumoral heterogeneity (ITH), genomic instability and therapy resistance [1,2,3]. It is conceivable that this triad has a major impact on patient survival [4,5,6].

One aspect of ITH that is especially understudied is the interplay of functional and spatial ITH, i.e., the topological distribution of tumor cells with certain functional properties within the three-dimensional ecosystem of a tumor.

It has previously been shown that the tumor periphery in ccRCC is a spatial niche with a number of unique functional characteristics [7]. This includes tumor cell proliferation, which shows maximum activity in the tumor periphery [7]. The biological basis for this observation is poorly understood. A previous study showed no genomic alterations that could confer a proliferative advantage for tumor cells occupying the tumor peripheral niche [7]. However, there is experimental evidence to suggest that growth factors derived from the peritumoral microenvironment can stimulate RCC cell proliferation [8]. In addition, an “empty site” model has been proposed in which tumor cells proliferate more when surrounded by non-neoplastic cells or extracellular matrix [9]. In striking contrast, there are examples of tumor entities such as prostate and thyroid cancer in which the tumor periphery is not a hotspot for proliferation but rather for cellular senescence, i.e., a stable withdrawal from the cell division cycle [10,11]. There is also evidence for spatial differences in the immunological landscape of ccRCC that may contribute to intratumoral niche formation [12,13,14,15]. More comprehensive analyses of the intratumoral and peripheral niches in ccRCC are needed in order to better understand disease progression.

There are a number of methods to interrogate ITH including, but not limited to, multiplex immunofluorescence or immunohistochemistry, imaging mass cytometry, spatial transcriptomics and multiregional single-cell sequencing [16,17,18,19,20,21]. Digital spatial profiling (DSP) combines high-plexed analysis of protein or mRNA expression with an exceptionally versatile selection of regions of interest (ROIs). The technique uses oligonucleotide barcodes attached to antibodies or RNA probes via a UV-cleavable linker. Targeted UV illumination of ROIs enables the collection and enumeration of the cleaved oligonucleotides, thereby allowing a spatially resolved analysis of gene expression. Importantly, the selection of ROIs based on morphology markers permits the analysis of tissue landmarks or subsets of malignant or non-malignant cells [22,23].

In the present study, we have used DSP of protein expression to further characterize the tumor periphery and center in ccRCC. We identified the tumor periphery as a highly active biological niche with an upregulation of various signaling nodes and a unique cellular contexture that has an impact on disease progression.

## 2. Patients and Methods

### 2.1. Patient Samples and Targeted Next-Generation Sequencing (NGS)

In this proof-of-concept study, formalin-fixed, paraffin-embedded (FFPE) tissue sections from 17 consecutive patients with ccRCC were analyzed (Table 1). Eleven samples were collected from primary tumors (64.7%), one sample from a local recurrence (5.9%) and five tissue samples from metastatic lesions (29.4%).

FFPE tissue sections were retrieved from the tissue bank of the National Center for Tumor Diseases (NCT) Heidelberg. All tissue-based experiments in this study were in accordance with the regulations of the tissue bank as well as under the approval of the Ethics Committee of the University of Heidelberg School of Medicine (206/2005, 207/2005, S-864/2019).

The tumor mutational status was determined with targeted panel sequencing using the capture-based TruSight™ Oncology 500 panel (Illumina, Cambridge, UK). This gene panel includes all relevant putative driver genes for RCC and covers 523 genes (full exonic coverage) as described previously [15]. Targeted NGS was performed under the approval of the Ethics Committee of the University of Heidelberg School of Medicine after written informed consent of the patient was obtained.

### 2.2. Digital Spatial Profiling

The GeoMx^®^ DSP platform (NanoString Technologies, Seattle, WA, USA) was used for the spatial analysis of ccRCC samples [22].

Briefly, 5 µm FFPE tissue sections on glass slides were deparaffinized and rehydrated (3 × 5 min xylene, 2 × 5 min each in 100% ethanol, 95% ethanol and ddH_2_O). Antigens were retrieved by boiling slides in 1x citrate buffer (pH 6.0) for 15 min using a pressure cooker. Sections were washed in TBS-T and blocked for 1 h in a humidified chamber at room temperature (RT) using Buffer W (NanoString). Tissue sections were then incubated overnight at 4 °C in a humidified chamber with the GeoMx^®^ PI3K/AKT (9 protein targets) and MAPK (10 protein targets) signaling assays, the GeoMx^®^ Cell Death assay (10 protein targets), as well as the GeoMx^®^ Immune Cell Profiling Core assay (18 protein targets; all NanoString), which also includes three barcode-labeled secondary antibodies (rabbit IgG, mouse IgG1 and IgG2a) as negative controls and antibodies against three housekeeping proteins (histone H3, GAPDH, ribosomal protein S6; Table 2).

To enable the identification of spatial landmarks and general tissue morphology, samples were stained for pan-cytokeratin (epithelial cells) and CD45 (immune cells) using the Solid Tumor TME Morphology Kit (catalog #121300301, NanoString).

After antibody incubation, sections were washed in TBS-T (3 × 10 min) and fixed with 4% paraformaldehyde for 30 min (RT). After washing in TBS-T (2 × 5 min), nuclei were stained with 500 nM SYTO13 (NanoString) for 15 min (RT) followed by a final wash in TBS-T. Slides were either directly loaded onto the GeoMx^®^ instrument (v2.4.2.2) or covered with Fluoromount-G^®^ (SouthernBiotech, Birmingham, AL, USA) and a coverslip and stored at 4 °C until processing. Following slide scanning, representative circular or polygonal ROIs were selected by a trained pathologist (S.D.) in the tumor periphery directly adjacent to the surrounding stroma and in the tumor center. Before ROI selection, hematoxylin- and eosin-stained tissue sections were examined to identify suitable areas for ROI placement. ROIs in each niche were placed to represent different cellular contextures including immune cell infiltration. Areas of necrosis or hemorrhage were excluded.

After the UV illumination of ROIs, collected barcodes were hybridized with fluorescent probes according to the manufacturer’s protocol (16 h at 67 °C). Hybridized barcodes were purified with the nCounter^®^ MAX/FLEX Prep Station (v4.1.0.1; NanoString) and counted on the nCounter^®^ Digital Analyzer (v4.0.0.3; NanoString). Counts were then transferred to the GeoMx^®^ instrument and normalized to the geometric mean of all three housekeeping proteins. To identify statistically significant differences in spatial protein expression, a linear mixed model (LMM) analysis [24] was used after a log10 transformation of counts to test for expression differences of proteins between tissue sites (fixed effect), considering that several ROIs were taken from the same specimen (random effect). To take multiple testing into account, resulting *p* values were corrected after the Benjamini-Hochberg method to control the false discovery rate and were displayed as a volcano plot.

### 2.3. Immunohistochemical Staining

FFPE tissue sections were deparaffinized in xylene and rehydrated in a graded ethanol series. Antigen retrieval was performed using antigen-retrieval buffer (Dako, Glostrup, Denmark), heated in a steamer. Tissue sections were incubated with a primary antibody directed against granzyme B (EPR8260, 1:100; Abcam, Cambridge, UK). For immunodetection, tissue specimens were incubated with a biotinylated secondary goat anti-rabbit IgG antibody (ab97049, 1:200; Abcam) and streptavidin–peroxidase conjugate (Merck/Sigma-Aldrich, Taufkirchen, Germany). Staining was detected using the DAB Substrate Kit (Abcam), and nuclei were visualized with Hematoxylin Gill I (Sigma-Aldrich, St. Louis, MO, USA) before mounting. The numbers of granzyme B-positive cells per 20× microscopic field were counted manually. At least three 20× microscopic fields were counted per sample. The 20× fields were randomly chosen and did not correspond to the ROIs selected for DSP.

### 2.4. Statistical Analysis and R Packages

To identify statistically significant differences in spatial protein expression, a LMM analysis with *p* value correction was used. Differences in the distribution of clinico-pathological parameters were calculated using Fisher’s Exact test. The Mann–Whitney U test was employed to test for differences in granzyme B expression. The IBM SPSS software v. 27 was used for Kaplan–Meier curves. A *p*-value of ≤0.05 was considered statistically significant. R packages used for visualization and LMMs were tidyverse (2.0.0), ggpubr (0.6.0), ComplexHeatmap (2.14.0), emmeans (1.8.5) and the lme4 package (1.1.32) [24].

## 3. Results

### 3.1. DSP of Protein Expression Identifies the Tumor Periphery as an Intratumoral Niche with Upregulation of Various Signaling Nodes and a Unique Cellular Contexture

**Figure 1:**
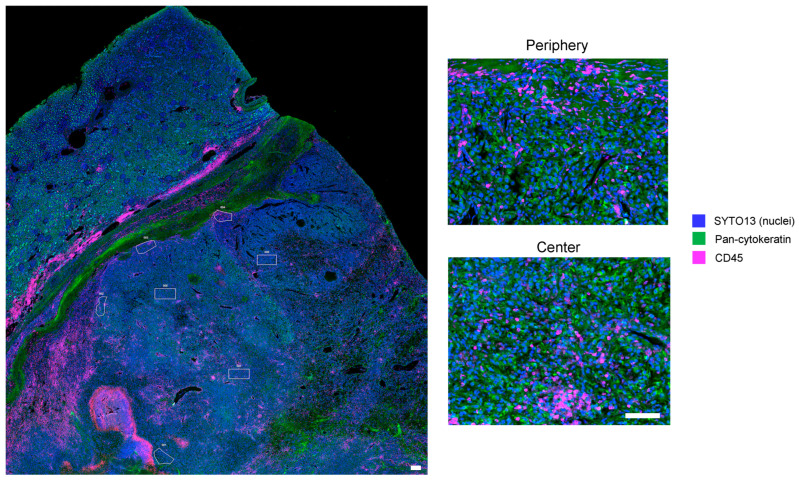
DSP of protein expression of archival ccRCC specimens to compare tumor periphery and center. Representative GeoMx® DSP scan of an archival FFPE ccRCC specimen following staining for the morphology markers CD45 (red) and pan-cytokeratin (green). Nuclei are stained with SYTO13 (blue). Rectangular and polygonal ROIs (white) in the tumor periphery and the tumor center are shown. Higher magnification images from the tumor periphery and the tumor center (right panels) are shown to illustrate proper staining of the morphology markers in the two areas. Scale bars = 100 μm.

A total of 128 ROIs (67 from the tumor periphery and 61 from the tumor center) from 17 ccRCC specimens were analyzed (Figure 1). Overall, the average number of ROIs per tumor specimen was 7.5 (range, 4 to 13 ROIs). The average number of ROIs collected was 3.9 (range, 2 to 9 ROIs) in the tumor periphery and 3.6 (range, 2 to 7 ROIs) in the tumor center. Overall, the average ROI surface area was 0.093 mm^2^ (range, 0.012 to 0.23 mm^2^). The average ROI surface area in the tumor periphery was 0.077 mm^2^ (range, 0.012 to 0.23 mm^2^) and 0.111 mm^2^ (range, 0.022 to 0.209 mm^2^) in the tumor center. 

**Figure 2:**
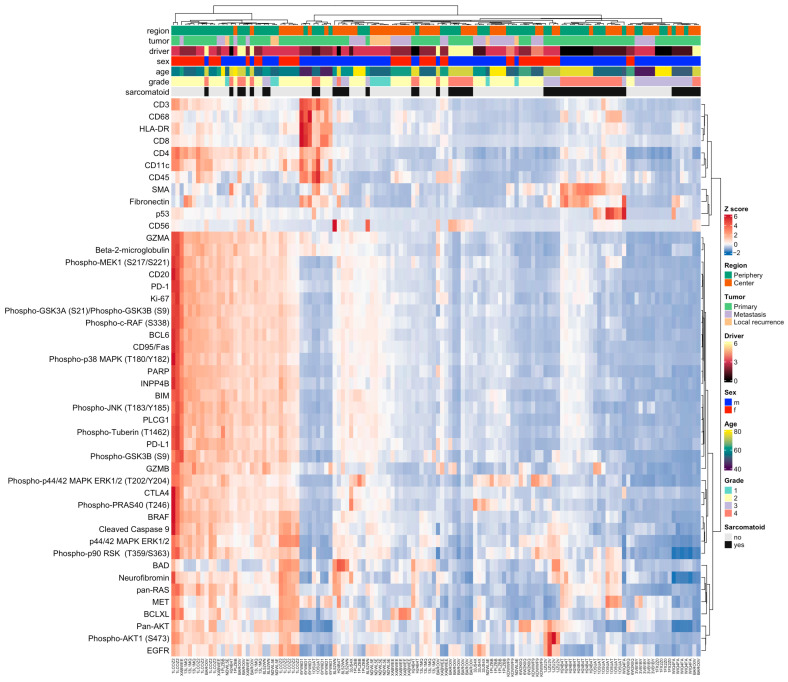
DSP identifies two ccRCC protein expression clusters. Unsupervised clustering and heatmap of DSP protein expression data obtained from 17 patients and 128 ROIs. For clustering, protein expression values were z-transformed by subtracting the protein mean from each protein and dividing by its standard deviation. Results are presented according to the color scale. “Driver” denotes the number of high-confidence driver gene mutations in analogy to [1] ranging from 0 to 6.

Unsupervised hierarchical cluster analysis revealed two major protein expression clusters (Figure 2). Cluster 1 showed predominantly an upregulation of protein expression, while cluster 2 was largely characterized by a downregulation or no change of protein expression. ROIs in cluster 1 were found to localize predominantly to the tumor periphery (77%), whereas ROIs in cluster 2 showed a more balanced association with tumor regions (44% tumor periphery vs. 56% tumor center). The differences between the two clusters in terms of ROI localization were statistically significant (*p* = 0.002, Fisher’s Exact test). 

Cluster 1 contained more ROIs from tumors with three or more driver gene mutations (*p* = 0.007), less ROIs from tumors with sarcomatoid differentiation (*p* = 0.04) and more ROIs from tumors with a histological grade 2 but less from grade 3 or 4 tumors (*p* = 0.02) compared to cluster 2.

**Figure 3:**
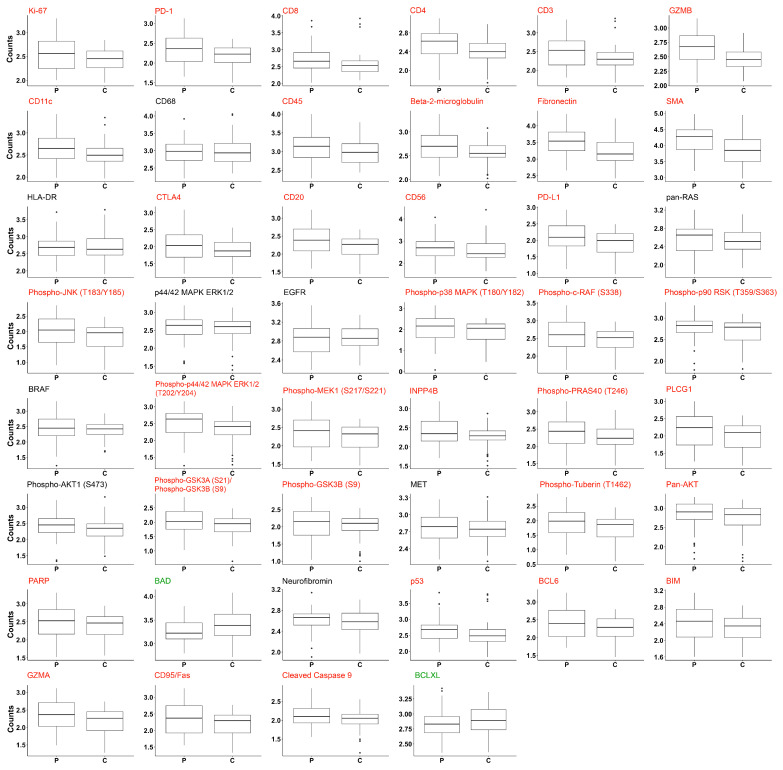
Tumor periphery and center are characterized by a high number of differentially expressed proteins. Box plots showing the expression of 46 proteins in the tumor periphery (P) and the tumor center (C). LMM analysis of log10 transformed counts was used to assess statistically significant differences. Proteins significantly upregulated in the tumor periphery are highlighted in red. Only two proteins (BAD and BCLXL) were significantly upregulated in the tumor center (green).

A LMM analysis revealed that 37 of the 46 proteins analyzed were differentially expressed between the tumor periphery and the tumor center (Figure 3). 

All but two of the differentially expressed proteins were found to be upregulated in the tumor periphery. Among the proteins that were significantly upregulated in the tumor periphery were Ki-67 and components of the PI3K/AKT/mTOR signaling network such as phospho-GSK3A (S21)/phospho-GSK3B (S9), INPP4B, PLCG1, phospho-PRAS40 (T246), phospho-Tuberin (T1462) and phospho-GSK3B (S9), which confirms previous findings [7]. In addition, proteins involved in MAPK signaling including phospho-p44/42 ERK1/2 (T202/Y204), phospho-JNK (T183/Y185), phospho-p38 MAPK (T180/Y182), phospho-c-RAF (S338), phospho-p90 RSK (T359/S363), phospho-MEK1 (S217/S221), immune responses (granzyme B/GZMB, CD4, PD-L1, PD1, CD20, beta-2-microglobulin, CD11c, CD3, CD8, CTLA4, CD45, CD56), apoptosis regulation (CD95/Fas, BCL6, BIM, PARP, GZMA, p53), and epithelial-to-mesenchymal transition (EMT; SMA, fibronectin) were significantly upregulated. 

**Figure 4:**
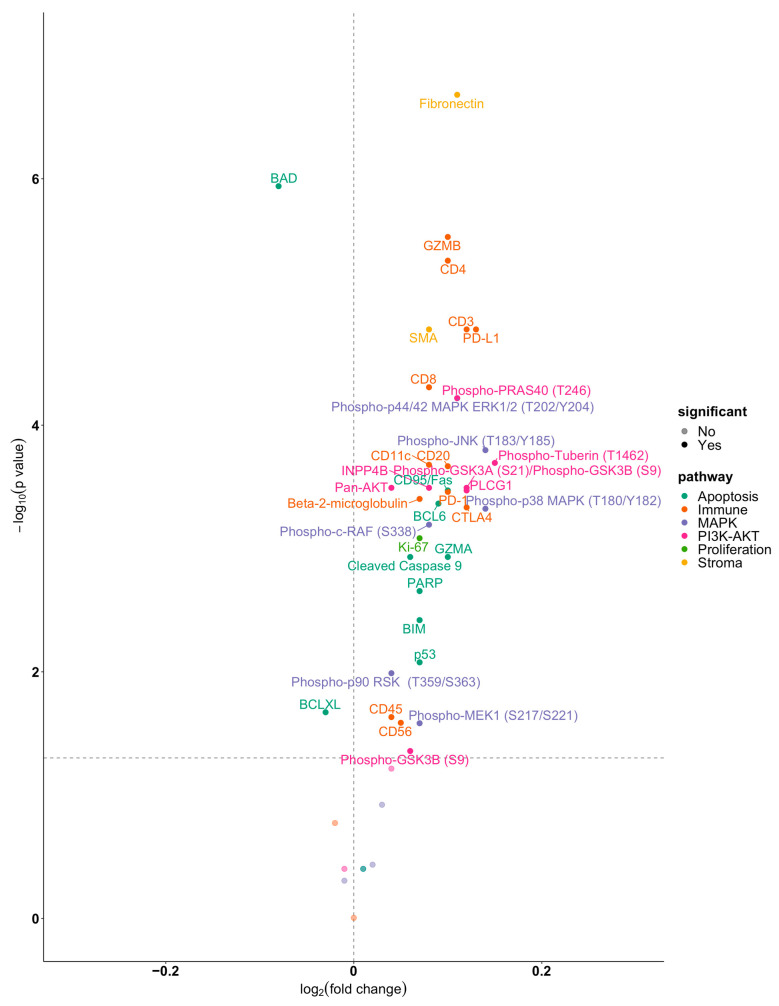
DSP identifies the tumor periphery as a highly active biological niche with upregulation of functionally diverse proteins. The volcano plot depicts differentially expressed proteins between the tumor periphery and the tumor center. Proteins with a false discovery rate corrected *p* ≤ 0.05 are represented. Colors represent functionally related groups of proteins.

The most significantly upregulated proteins in the tumor periphery were fibronectin and granzyme B as shown in the volcano plot in Figure 4. In contrast, BAD and BCLXL were the only proteins found to be upregulated in the tumor center (Figure 4). A LMM analysis was used to test protein expression in tissue specimens from different origins, i.e., primary tumor, local recurrence or metastasis. None of the 46 proteins analyzed were found to be differentially expressed. 

Taken together, these results confirm and extend previous findings and highlight the tumor periphery as a highly active spatial niche with unique biological features.

### 3.2. Granzyme B Upregulation Characterizes the Tumor Periphery in a Subset of ccRCCs and Correlates with Disease Progression

**Figure 5:**
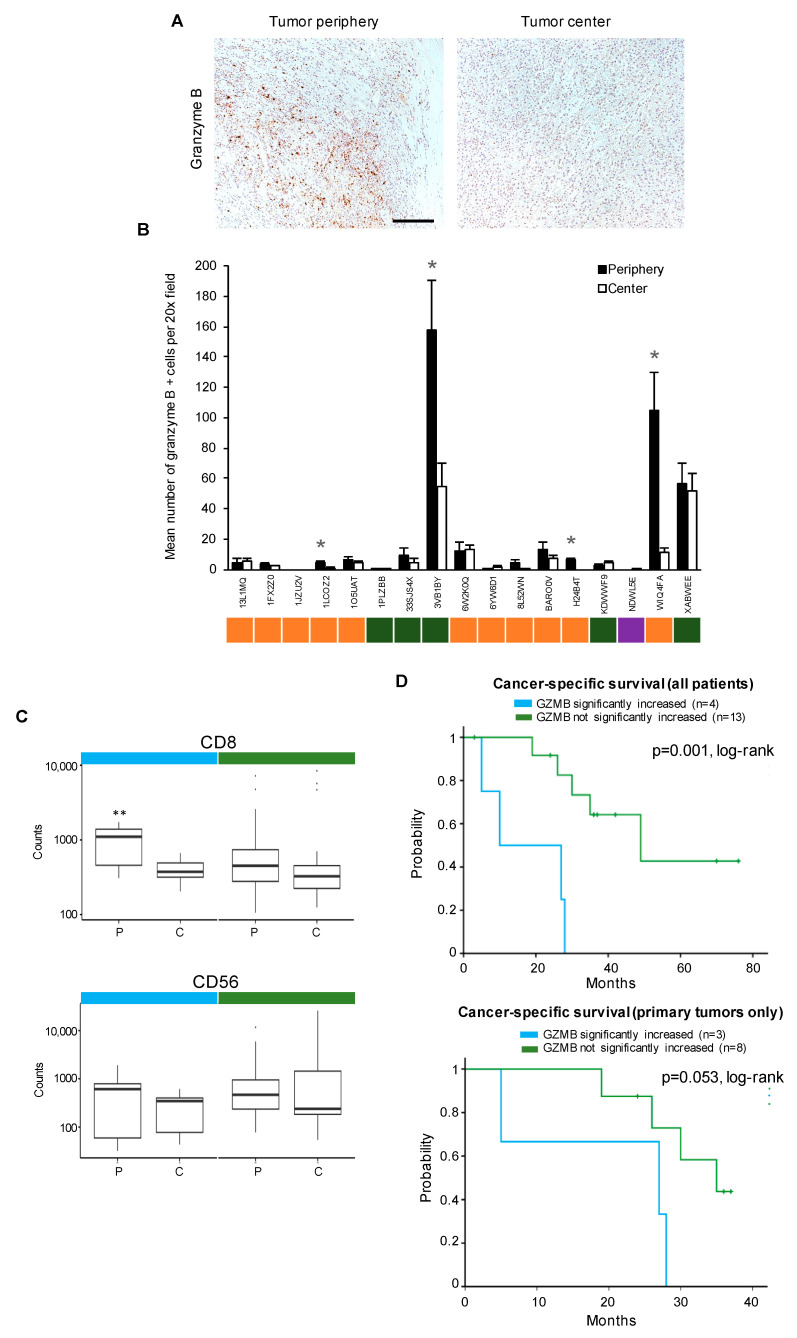
Upregulation of granzyme B in the tumor periphery is associated with a poor cancer-specific patient survival. (**A**) Immunohistochemical staining for granzyme B. Representative microphotographs from the tumor periphery and the tumor center are shown. Scale bar = 250 µm. (**B**) Quantification of the number of granzyme B-positive cells per 20× microscopic field in the tumor periphery and the tumor center. Each bar shows mean and standard error of at least three 20× fields counted. Colors represent the origin of the respective tissue specimen (orange, primary tumor; purple, local recurrence; green, metastasis). *, *p* ≤ 0.05. (**C**) Analysis of DSP data for CD8 (top) and CD56 (bottom) expression in the tumor periphery (P) versus tumor center (C) for the four patients with significantly upregulated granzyme B expression in the tumor periphery (turquoise) in comparison to patients with no significant upregulation (green). **, *p* ≤ 0.005. (**D**) Kaplan–Meier curves of cancer-specific survival of all patients (*n* = 17, upper panel) or only patients for which specimens derived from the primary tumor were analyzed (*n* = 11, lower panel) stratified into patients with significantly upregulated granzyme B expression in the tumor periphery and patients with no significant upregulation.

One of the most significantly upregulated proteins in the tumor periphery was granzyme B. In order to validate the differential expression of granzyme B in the tumor periphery in comparison to the tumor center, we stained all samples for granzyme B using conventional immunohistochemistry (Figure 5). While some tumors showed a high number of granzyme B-positive cells in the tumor periphery (Figure 5A), most ccRCCs did not follow this pattern. To assess the intertumoral differences in granzyme B expression, the number of granzyme B-positive cells per 20× microscopic field was determined. In 4 of 17 ccRCC specimens, there was a significantly higher expression of granzyme B-positive cells in the tumor periphery when compared to the tumor center (*p* ≤ 0.05; Figure 5B).

However, the majority of tumors showed no statistically significant differences between the two compartments, and the number of granzyme B-positive cells overall was low. Interestingly, the sample with the highest number of granzyme B-positive cells (3VB1BY) was derived from a liver metastasis.

In an attempt to identify the cellular origin of granzyme B in the four cases with significantly upregulated expression, we separately analyzed the DSP data of these tumors. We found a statistically significant upregulation of CD8 (*p* ≤ 0.005) but not CD56 expression in these tumors (Figure 5C). We hasten to add that this analysis does not prove a correlation between the cellular contexture and granzyme B expression.

Lastly, we sought to explore whether a significantly increased expression of granzyme B in the tumor periphery correlates with disease progression. Remarkably, a significantly increased expression of granzyme B in the tumor periphery was associated with a significantly worse cancer-specific survival (CSS) of patients (*p* = 0.001, Figure 5D, upper panel). A separate analysis of the CSS of patients whose primary tumors were examined showed a trend toward a worse survival outcome (*p* = 0.053, Figure 5D, bottom panel). 

In conclusion, these results highlight the intertumoral heterogeneity of granzyme B expression with low or absent expression in the majority of ccRCCs and a high expression in the tumor periphery in a subset of tumors. Of note, a high expression of granzyme B in the tumor periphery was associated with a worse CSS suggesting accelerated disease progression.

## 4. Discussion

Genomic ITH is a well-known characteristic of ccRCC [1,2,25]. In contrast, ITH on the functional and spatial levels is much less understood. Previous studies have shown that the tumor periphery in ccRCC harbors proliferating tumor cells that show activated survival signaling [7]. Whether and to what extent other molecular alterations that can drive tumor growth and disease progression are enriched in the tumor periphery is unknown.

In the present study, we used DSP of protein expression to interrogate the tumor periphery in ccRCC in greater detail. Out of 46 proteins examined, 35 were upregulated in the tumor periphery compared to the tumor center. These included the proliferation marker Ki-67 and components of the PI3K-AKT signaling pathway but also proteins involved in apoptosis regulation, MAPK signaling, EMT as well as immune cell markers. 

One of the most significantly upregulated proteins in the tumor periphery was granzyme B. Validation experiments showed intertumoral heterogeneity of granzyme B upregulation. We could demonstrate that upregulation of granzyme B in the tumor periphery was associated with disease progression resulting in a significantly worse CSS of ccRCC patients. These findings underscore the importance of the tumor periphery as a functional niche in ccRCC that may contribute to aggressive tumor growth and may hence also convey prognostic information.

Although the exact mechanisms leading to the formation of the peripheral niche, such as growth factors produced by adjacent stroma cells [8] or altered physical restraints (“empty site” model [9]), remain to be determined, our results nevertheless suggest that the tumor periphery is a hotspot for interactions between tumor cells and the microenvironment. In fact, a co-evolution between tumor cells and their microenvironment has previously been proposed [26], and results shown here suggest that this may occur preferentially in the peripheral tumor niche. In silico modeling of ccRCC has suggested that tumor growth limited to the tumor periphery may be associated with higher subclonal diversification and enhanced genomic ITH [27]. Remarkably, an association between genomic ITH and composition of the tumor microenvironment that was mediated by tumor-derived cytokines has been reported [28]. The latter finding lends further support to the idea of co-evolution between tumor cells and the microenvironment. Whether the tumor periphery may in fact be the origin of metastasizing ccRCC clones [27] remains to be tested experimentally.

The finding that granzyme B was among the most significantly upregulated proteins in the ccRCC periphery was somewhat perplexing. Granzyme B is a protease expressed mainly by cytotoxic CD8+ T cells and NK cells. After being released from cytotoxic granules, it functions as an effector protein that—in cooperation with the pore-forming protein perforin—contributes importantly to tumor cell eradication [29]. However, in ccRCC, a high number of CD8+ tumor-infiltrating lymphocytes (TILs) has been reported to be associated with a poor prognosis, which is in contrast to many other tumor entities [30]. Most of these CD8+ TILs in ccRCC are exhausted, i.e., they are not capable of killing tumor cells, and one mechanism for this exhaustion phenotype is the loss of the effector protein granzyme B [31]. We could show with conventional immunohistochemistry that a significant upregulation of granzyme B in the tumor periphery was limited to a relatively small subset of tumors, while the majority may in fact represent tumors with mostly exhausted T cells. Moreover, tumors with a significantly increased granzyme B expression in the periphery based on conventional immunohistochemistry were not the highest expressing tumors using DSP. Since we did not analyze the same DSP ROIs with immunohistochemistry, this finding highlights not only the technical differences between the two methods but also points to a possible sampling bias. A rigorous independent validation of DSP results is therefore of paramount importance.

Although granzyme B is essential for the induction of tumor cell death by cytotoxic T or NK cells, and a low expression may hence impair the host immune response to tumor cells, an overexpression likewise may have negative consequences on patient prognosis as suggested by our results. This finding is difficult to reconcile with the antitumoral effector function of granzyme B in cytotoxic lymphocytes. However, the fact that all four patients with significantly increased granzyme B in the tumor periphery showed rapid disease progression and succumbed to ccRCC may point to the development of aggressive, apoptosis-resistant subclones under selection pressure from a high number of non-exhausted T cells. Similar findings have been reported in certain lymphomas and nasopharyngeal carcinoma [32,33,34]. Whether patients showing such a constellation may benefit more from immune checkpoint blockade than patients in which T cells are mostly exhausted remains to be determined [35]. 

It is noteworthy that cluster 2 contained a sub-cluster with an upregulation of a number of immune cell markers including CD3 and CD8. It has previously been shown that an immunoscore that ascertains expression of these immune markers in the tumor center and the invasive margin conveys prognostic information in colorectal cancer [36]. Whether such prognostic information can also be obtained in RCC remains to be shown. We would like to reiterate in this context that a high immune infiltration in RCC is, in contrast to other malignancies, associated with a worse prognosis since CD8+ T cells are commonly dysfunctional or terminally exhausted [30].

Previous studies have successfully used the GeoMx^®^ DSP platform to identify predictive and prognostic biomarkers in the tumor as well as the stroma compartment. Examples include studies in head and neck squamous cell carcinoma [37], non-small cell lung cancer [38], triple-negative breast cancer [39,40,41] and ccRCC [42]. In these tumor entities, immune checkpoint blockade is used for the systemic treatment of patients [43]. In the present study, the tumor microenvironment was not the initial focus. However, in light of these previous findings, further studies using GeoMx^®^ DSP for the discovery of prognostic and predictive biomarkers with a focus on the tumor microenvironment are warranted.

Limitations of the present study are the small sample size and the single-cohort nature. Nevertheless, DSP, even when it entails only a relatively small number of tumors, allows to identify expression signatures that are ideally suited for hypothesis-generating studies, such as ours.

## 5. Conclusions

Results of this proof-of-concept study highlight the tumor periphery as a highly active intratumoral niche that may contribute to disease progression and may hence also convey prognostic information for future patient stratification. 

## Figures and Tables

**Table 1:** Clinico-pathological patient characteristics.

Patient Characteristics (*n* = 17)	
Sex, m/f, *n*	11/6
Age, years, median (range)	60 (43–82)
c/p/yTNM stage, *n* (%) *	
T1	1 (5.9)
T2	3 (17.6)
T3	11 (64.7)
T4	0 (0)
Tx	2 (11.8)
N0	8 (47.1)
N1	4 (23.5)
Nx	5 (29.4)
M0	6 (35.3)
M1	8 (47.1)
Mx	3 (17.6)
Fuhrman Grade, *n* (%)	
1	1 (5.9)
2	8 (47.1)
3	5 (29.4)
4	3 (17.6)
Histology, *n* (%)	
Clear Cell	17 (100)
Tissue origin, *n* (%)	
Primary tumor	11 (64.7)
Local recurrence	1 (5.9)
Metastatic lesion	5 (29.4)
Bone	1 (20)
Brain	2 (40)
Liver	1 (20)
Skin	1 (20)

* for all patients (including TNM stage at the time of diagnosis for patients with advanced disease at the time of tissue analysis).

**Table 2:** Overview of Nanostring GeoMx^®^ DSP protein panels.

Immune Cell Profiling	Cell Death	MAPK Signaling	PI3K/AKT Signaling
Beta-2-microglobulin	BAD	EGFR	Pan-AKT
CD11c	BCL6	pan-RAS	MET
CD20	BCLXL	BRAF	Phospho-AKT1 (S473)
CD3	BIM	Phospho-c-RAF (S338)	Phospho-GSK3B (S9)
CD4	CD95/Fas	Phospho-JNK (T183/Y185)	Phospho-Tuberin (T1462)
CD45	GZMA	Phospho-MEK1 (S217/S221)	Phospho-GSK3A (S21)/Phospho-GSK3B (S9)
CD56	p53	Phospho-p38 MAPK (T180/Y182)	INPP4B
CD68	PARP	Phospho-ERK1/2 (T202/Y204)	PLCG1
CD8	Cleaved Caspase 9 Neurofibromin	ERK1/2 Phospho-p90 RSK (T359/S363)	Phospho-PRAS40 (T246)
CTLA4			
Pan-cytokeratin			
Fibronectin			
GZMB			
HLA-DR			
Ki-67			
PD-1			
PD-L1			
SMA			
Ms IgG2a			
Ms IgG1			
Rb IgG			
Histone H3			
S6			
GAPDH			

## Data Availability

The data presented in this study are available within the article.
